# The Effect of Dietary Peach Pomace on the Performance, Blood Parameters, Carcass Traits, Organ Weights, and Meat Quality Parameters of Broiler Chickens

**DOI:** 10.1002/fsn3.70770

**Published:** 2025-08-19

**Authors:** Tshegofatso Moroka, Kgaogelo Phineas Meso, Lebogang Ezra Motsei, Chidozie Freedom Egbu

**Affiliations:** ^1^ Department of Animal Science Faculty of Natural and Agricultural Science, North‐West University Mmabatho South Africa; ^2^ Food Security and Safety Faculty of Natural and Agricultural Sciences, North‐West University Mmabatho South Africa; ^3^ Department of Agricultural Science Faculty of Vocational and Technology Education, Alvan Ikoku Federal University of Education Owerri Nigeria

**Keywords:** alternative feed ingredient, biochemistry, nutraceutical, physiology, production, sustainability

## Abstract

Using peach ( *Prunus persica* L.) pomace as feed material could reduce the environmental impact of agro‐waste disposal while exploring alternative feed ingredients that may support sustainability in poultry production. Nonetheless, their nutritive value in poultry production remains unexplored. The effect of peach pomace meal (**PPM**) on broiler chickens' growth performance, physiological status, and meat quality was investigated. Seven‐day‐old male Ross 308 chicks (*n* = 280; 140 ± 0.07 g live‐weight) were randomly distributed to four experimental diets containing PPM at 0 (**CON**), 100 (**PPM100**), 150 (**PPM150**), and 200 g/kg (**PPM200**) in a standard broiler chicken diet. Each experimental diet comprising starter (7–14 days), grower (14–28 days), and finisher phases (28–35 days) was randomly assigned to 7 replicate pens (experimental units) with 10 birds each. Feed intake was higher (*p <* 0.05) in birds reared on PPM200 than in those fed CON during days 7–14 and 28–35. The body weight gain and feed conversion ratio among treatments did not vary (*p >* 0.05). The CON had a higher (*p <* 0.05) lymphocyte count and bilirubin concentration than the PPM groups. The PPM100 had higher (*p <* 0.05) breast meat redness than the CON and PPM150. Dietary PPM did not compromise growth, physiological status, or meat quality, implying that peach pomace meal could serve as a valuable feed ingredient in poultry production.

## Graphical abstract

Assessed peach pomace meal as an alternative feed ingredient for broilers. Tested dietary peach pomace at 0, 100, 150, and 200 g/kg in broiler diets. Broiler chicken weight gain, feed efficiency, or meat quality was not compromised. Dietary peach pomace meal reduced lymphocyte counts and bilirubin concentration. Promotes the valorization of fruit industry by‐products in animal nutrition by reducing indiscriminate disposal of agro waste.

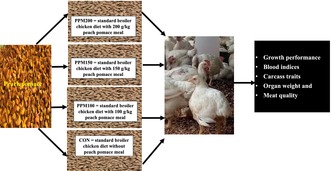

## 1 Introduction

Chicken meat is recognized for its high‐quality protein composition with increasing global consumer demand (Kleyn and Ciacciariello 2021; Barbut and Leishman 2022). The global poultry business yearly generates more than 9 trillion kg of chicken meat (Agyare et al. 2018). Moreover, it is anticipated that poultry meat will experience the most significant growth, estimated at 121% by 2050 (Govoni et al. 2021) given the exigency to satisfy the protein demand of the growing human population (Erinle and Adewole 2022). However, high feeding costs, comprising over 70% of the primary expenses in poultry production, negatively impact the poultry industry's economic sustainability (Mallick et al. 2020; Wongnaa et al. 2023). Thus, the economic and environmental sustainability of the poultry industry could rely significantly on using inexpensive and locally available feed materials.

Fruit by‐products such as olive pomace (Hlatshwayo et al. 2023), apple pomace (Matabane et al. 2024), and grape pomace (Thema et al. 2024) possessing beneficial bioactive compounds, can be integrated into poultry diets to enhance sustainable practices in animal agriculture. Peach pomace (PP) refers to the remnant seeds, skin, and stems after peach crushing in concentrates and juice‐making facilities. The amount of PP generated during processing varies depending on the ripeness of the peaches, with approximately 10% being lost (Argun and Dao 2017). Traditional disposal methods of fruit by‐products, such as landfilling or incineration, have severe environmental adversities, such as land and water pollution (Esparza et al. 2020) and carbon dioxide and methane release into the environment (Chavan et al. 2022). Annually, over 200 000 tons of PP are produced in South Africa; hence, utilizing them in chicken feeds could help mitigate waste and convert an environmental burden into a valuable nutritional resource. This strategy can potentially reduce the ecological footprint of the agricultural and food processing industries (Jonathan et al. 2021).

Nutritionally, PP contains 25.9% water‐soluble carbohydrates, 7.36% crude protein, 1.99 mg/100 g catechin, and 1.31 mg/100 g chlorogenic acid (Hu et al. 2015; Vulić et al. 2022), which can be beneficial in poultry nutrition. However, studies reported varying outcomes for growth performance, physiological status, and meat quality when fruit pomaces were supplemented as nutraceuticals in poultry diets (Akuru et al. 2020; Hlatshwayo et al. 2023; Kumanda et al. 2019; Matabane et al. 2024). Although PP is primarily incorporated to utilize its nutraceutical benefits and reduce environmental waste, its high 20% neutral detergent fiber content (Hu et al. 2015) may alter the overall digestibility of the diet. This change in digestibility could potentially impact growth performance, particularly at higher inclusion levels.

Beyond pomace, other peach processing residues have shown promise in animal nutrition. Peach kernel extract has been evaluated as a dietary feed additive for growing rabbits, improving weight gain, feed conversion ratio, and immune status (Basyony et al. 2023). Interestingly, to the authors' knowledge, no research has evaluated the feeding value of peach pomace meal (PPM) in poultry. Thus, the current investigation determined the effect of incremental PPM levels in broiler chicken diets on growth performance, physiological status, and meat quality.

## 2 Materials and Methods

### 2.1 Ethics Approval and Experimental Location

Study procedures, animal care, rearing, and slaughtering adhered to experimental animal care guidelines approved (NWU‐0029‐23‐A5) by the Animal Production Research Ethics Committee of North‐West University, Mafikeng, South Africa.

The study took place between November and December 2023 at the North‐West University research farm (Molelwane; 25°34′58″ S, 25°64′32″ E), South Africa.

### 2.2 Feed Ingredient Sources and Preparation

Fresh, wet PP was obtained from The DELI, Bellville, Western Cape, South Africa, and was dried in an EcoTherm Digital oven (LABOTEC, Midrand, South Africa) at 60°C until reaching a constant weight before being finely ground to a particle size of 2 mm using a cutting mill (SM 100, Retsch GmbH, Haan, Germany) to produce PPM. All additional feed components utilized in this study were obtained from SimpleGrow Agric Services (PTY) LTD (Centurion, Gauteng, South Africa). Four experimental diets (Table 1) with similar energy and protein content were produced to fulfill the nutritional requirements of Ross 308 broiler chickens (Aviagen, 2018). The PPM was incorporated into standard broiler starter crumb, grower pellet, and finisher pellet diets at levels of 0 (CON), 100 (PPM100), 150 (PPM150), and 200 g/kg (PPM200).

**TABLE 1:** Gross composition of the experimental diets (g/kg as fed) containing peach pomace meal.

	Treatments^a^											
	Starter (7–14 days)				Grower (14–28 days)				Finisher (28–35 days)			
Ingredients	CON	PPM100	PPM150	PPM200	CON	PPM100	PPM150	PPM200	CON	PPM100	PPM150	PPM200
Peach Pomace	0.00	100.0	150.0	200.0	0.00	100.0	150.0	200.0	0.00	100.0	150.0	200.0
Maize Yellow	526.7	411.0	349.0	290.0	577.0	455.8	399.1	334.2	464.9	401.1	359.2	310.0
Pellibond^b^	10.0	10.0	10.0	10.0	15.0	15.0	15.0	15.0	10.0	10.0	10.0	10.0
Soya Full Fat	150.0	150.0	180.0	180.0	70.0	70.0	59.1	77.4	—	—	—	—
Soybean Meal (465 g/kg)	219.4	222.8	204.5	207.6	142.8	154.4	176.4	165.5	118.2	145.0	150.0	152.4
Sunflower Oilcake (340 g/kg)	15.0	15.0	15.0	15.0	65.0	66.0	56.7	60.9	80.0	84.5	90.0	95.7
Betta Fish	2.00	6.00	6.00	7.00	50.3	48.2	46.4	45.9	60.0	50.9	50.0	50.0
Wheat Bran	—	—	—	—	—	—	—	—	124.5	70.0	51.5	40.0
Berga Fat	3.00	7.30	5.00	10.0	11.2	18.7	21.2	23.0	74.3	69.9	65.4	67.7
Crude Soya Oil	25.0	30.0	33.0	33.0	26.0	30.0	34.0	36.0	25.0	25.0	30.0	30.0
L‐Lysine HCl	3.20	3.20	3.20	3.20	3.00	2.90	2.80	2.80	3.00	3.00	3.00	3.00
DL‐Methionine	1.70	1.80	1.80	1.90	1.30	1.40	1.50	1.30	1.90	1.90	1.90	1.90
L‐Threonine	1.40	1.50	1.50	1.60	0.70	0.80	0.90	0.90	0.80	0.80	0.80	0.80
Fine Feed Lime	24.4	23.4	22.8	22.3	23.1	22.2	22.0	22.0	25.5	25.5	25.5	25.5
MDCP^c^	8.70	8.50	8.70	8.90	5.70	5.70	6.00	6.20	3.80	4.20	4.50	4.80
Fine Salt	2.00	2.00	2.00	2.00	2.00	2.00	2.00	2.00	2.60	2.60	2.60	2.60
Sodium Bicarbonate	1.40	1.40	1.40	1.40	1.30	1.30	1.30	1.30	0.40	0.40	0.50	0.50
Choline Chloride	2.00	2.00	2.00	2.00	2.00	2.00	2.00	2.00	2.00	2.00	2.00	2.00
Avatec^d^	0.50	0.50	0.50	0.50	0.50	0.50	0.50	0.50	0.50	0.50	0.50	0.50
Zinc Bacitracin (150 g/kg)	0.50	0.50	0.50	0.50	0.50	0.50	0.50	0.50	0.50	0.50	0.50	0.50
AxtraPhy (1000 FTU/g)	0.10	0.10	0.10	0.10	0.10	0.10	0.10	0.10	0.10	0.10	0.10	0.10
Starter premix^e^	3.00	3.00	3.00	3.00	—	—	—	—				
Grower premix^f^	—	—	—	—	2.50	2.50	2.50	2.50	—	—	—	—
Finisher premix^g^	—	—	—	—	—	—	—	—	2.00	2.00	2.00	2.00

^a^ Treatments: CON = a standard broiler chicken diet without peach pomace meal; PPM100 = a standard broiler chicken diet with 100 g/kg peach pomace meal; PPM150 = a standard broiler chicken diet with 150 g/kg peach pomace meal; PPM200 = a standard broiler chicken diet with 200 g/kg peach pomace meal.

^b^ Pellibond: Pellet binders derived from lignin with outstanding efficacy.

^c^ MDCP: Mono dicalcium phosphate.

^d^ Avatec: Lasolacid (150 mg/kg).

^e^ Starter premix = vitamin B12 (0.012 mg); niacin (29.7 mg); vitamin E (25 IU); vitamin K (2.97 mg); folic acid (1.0 mg); choline (801 mg); biotin (0.3 mg); pyridoxine (4.9 mg); thiamine (2.9 mg); vitamin A (9750 IU); vitamin D3 (2000 IU); riboflavin (7.6 mg); zinc (80.0 mg); copper (25 mg); Dl Ca‐pantothenate (13.5 mg); manganese (70.2 mg); selenium (0.15 mg); ethoxyquin (50 mg); wheat middlings (1543 mg); ground limestone (500 mg).

^f^ Grower premix = vitamin D3 (2000 IU); vitamin K (2.97 mg); Dl Ca‐pantothenate (13.5 mg); vitamin A (9750 IU); vitamin E (25 IU); vitamin B12 (0.012 mg); niacin (29.7 mg); folic acid (1.0 mg); riboflavin (7.6 mg); choline (801 mg); biotin (0.3 mg); pyridoxine (4.9 mg); thiamine (2.9 mg); copper (25 mg); zinc (80.0 mg); manganese (70.2 mg); ground limestone (500 mg); selenium (0.15 mg); ethoxyquin (50 mg); wheat middlings (1543 mg).

^g^ Finisher premix = vitamin A (10,500 IU); vitamin D3 (2500 IU); vitamin E (40 mg); vitamin B1 (1.5 mg); vitamin B2 (5.5 mg); vitamin B6 (3.25 mg); vitamin B12 (25 mcg); vitamin K3 (2 mg); biotin (100 mcg); nicotinic acid (35 mg); cal‐pantothenate (12 mg); folic acid (1.0 mg); choline chloride (298.4 mg); manganese (80 mg); copper (8 mg); iron (60 mg); selenium (0.2 mg); iodine (0.8 mg); zinc (50 mg); cobalt (0.4 mg); ethoxyquin (50 mg); wheat middlings (1543 mg).

### 2.3 Nutritional Determination of Peach Pomace Meal and Experimental Diets

The PPM and experimental diets were analyzed for dry matter (method 930.15; EcoTherm Digital oven, LABOTEC, Midrand, South Africa), ash content (method 942.05; muffle furnace, Nabertherm, Lilienthal, Germany), crude protein (method 976.05; Kjeldahl apparatus Büchi K‐370, Flawil, Switzerland), starch (method 996.11; Double Beam UV–Vis spectrophotometer, Selectech, Johannesburg, South Africa), arginine, histidine, isoleucine, lysine, methionine, threonine, valine (method 982.30), and tryptophan (method 988.15; HPLC system (Agilent 1260 Infinity, Santa Clara, CA, USA) with fluorescence detector using AOAC (2005) procedures (Table 2)). The crude fiber (**CF**), nitrogen detergent fiber (**NDF**), and acid detergent fiber (**ADF**) were assessed utilizing the ANKOM^DELTA^ automated fiber analyzer (ANKOM Technologies, NY, USA). During the NDF measurement, alpha‐amylase was used following the manufacturer's guidelines. Acid detergent lignin (**ADL**) was evaluated by immersing cellulose from the ADF residue bags in 72% (w/w) concentrated sulfuric acid for 3 h. The ether extract was measured with an ANKOM^XT15^ extractor (ANKOM Technologies, NY, USA). The calcium, chloride, phosphorus, and sodium content analyses were conducted utilizing an XRF spectrometer (Epsilon 4) supplied by Malvern Panalytical (Pty) Ltd., Randberg, South Africa, following methods by AgriLASA (1998). Gross energy was assessed using a Gallenkamp Ballistic Bomb Calorimeter under the methodology outlined by Henken et al. (1986), and these data were utilized to compute metabolizable energy (**ME**). The concentration of total soluble phenolics (**TSP**) was measured using a Double Beam UV–Vis spectrophotometer (Selectech, Johannesburg, South Africa) according to the Folin–Ciocalteau method (Makkar 2003), and the results were presented as tannic acid equivalents (g TAE/kg). Calculating the total insoluble phenolics (**TiPh**) fraction involved utilizing the remnants of phenolic extraction by repeating the Folin–Ciocalteau method. Samples and standards were measured at 765 nm for the TSP and TiPh. The soluble condensed tannins (**SCT**) were assessed by employing a blend of 95% butanol and 5% concentrated hydrochloric acid (37% w/w) as described by Porter et al. (1985), and the results were expressed as absorbance units (**AU**) per 200 mg sample. Determining the insoluble condensed tannins (**iCT**) fraction involved butanol‐HCl reaction with the sample residue obtained from TSP extraction, as Makkar (2003) outlined. The SCT and iCT used a thermostatted water bath (WTB24, Memmert GmbH + Co.KG, Schwabach, Germany) for the heat reaction and absorbance read at 550 nm on the same Double Beam UV–Vis spectrophotometer.

**TABLE 2:** Analyzed nutritional composition of the experimental diets (g/kg DM, unless otherwise specified) containing peach pomace meal.

	Treatments^a^												
		Starter (7–14 days)				Grower (14–28 days)				Finisher (28–35 days)			
Composition	PPM^b^	CON	PPM100	PPM150	PPM200	CON	PPM100	PPM150	PPM200	CON	PPM100	PPM150	PPM200
Dry matter (g/kg)	882	886	898	905	911	886	899	905	912	892	903	909	915
Crude protein	106	215	215	214	214	199	199	200	199	185	184	184	185
Ash	63.6	40.1	39.5	39.2	38.9	35.1	34.8	34.6	34.4	34.4	33.4	32.9	32.6
Ether extract	86.0	80.2	86.1	90.3	93.6	78.4	85.9	88.5	93.7	127	119	117	118
Crude fiber	252	30.3	35.8	42.9	48.2	33.9	45.7	49.4	54.7	42.8	51.9	56.6	62.8
Starch	79.9	371	304	268	233	399	329	297	259	339	298	272	243
Neutral detergent fiber	469	145	149	155	160	166	171	177	183	180	185	187	190
Acid detergent fiber	335	103	116	120	124	115	121	128	133	141	145	151	156
Acid detergent lignin	194	28.5	34.3	38.2	39.6	45.2	46.3	47.5	49.6	48.3	50.2	54.5	57.4
Metabolizable energy (MJ/kg)	11.6	12.4	12.4	12.4	12.4	12.7	12.7	12.7	12.7	13.0	13.0	13.0	13.0
SCT^c^ (AU_550_ nm/200 mg)	1.23	0.04	0.13	0.21	0.27	0.05	0.12	0.22	0.29	0.06	0.17	0.28	0.33
iCT^d^ (AU_550_ nm/200 mg)	0.39	0.03	0.06	0.09	0.12	0.03	0.07	0.10	0.13	0.05	0.09	0.14	0.18
TSP^e^ (g TAE/kg)	15.8	0.29	1.70	2.55	3.39	0.25	1.79	2.75	3.77	0.31	1.87	2.95	3.87
TiPh^f^ (g TAE/kg)	4.43	0.06	0.64	0.99	1.32	0.11	0.68	1.02	1.35	0.10	0.67	1.00	1.34
Calcium	24.8	13.2	12.9	12.7	12.6	12.1	11.8	11.8	11.8	12.4	12.6	12.6	12.7
Phosphorus	3.21	7.33	7.17	7.06	7.00	6.67	6.56	6.49	6.43	6.90	6.58	6.45	6.35
Magnesium	1.96	1.62	1.54	1.53	1.49	1.48	1.42	1.36	1.35	1.58	1.46	1.41	1.38
Potassium	2.23	9.46	9.19	9.08	8.96	8.03	7.86	7.81	7.71	7.81	7.46	7.27	7.09
Chlorine	1.01	2.41	2.37	2.34	2.31	2.53	2.46	2.39	2.36	3.00	3.00	3.00	2.81
Lysine (g/100 g DM)	3.18	1.22	1.23	1.23	1.24	1.12	1.12	1.12	1.12	1.02	1.02	1.02	1.02
Methionine (g/100 g DM)	1.87	0.45	0.45	0.45	0.45	0.45	0.45	0.45	0.43	0.50	0.49	0.48	0.48
Cysteine (g/100 g DM)	5.14	2.79	2.67	2.61	2.56	2.31	2.23	2.19	2.15	2.09	2.05	2.01	1.96
Threonine (g/100 g DM)	12.2	0.80	0.80	0.80	0.80	0.71	0.71	0.71	0.70	0.66	0.65	0.65	0.64
Tryptophan (g/100 g DM)	1.27	0.21	0.21	0.21	0.21	0.19	0.18	0.19	0.18	0.18	0.17	0.17	0.17

^a^ Treatments: CON = a standard broiler chicken diet without peach pomace meal; PPM100 = a standard broiler chicken diet with 100 g/kg peach pomace meal; PPM150 = a standard broiler chicken diet with 150 g/kg peach pomace meal; PPM200 = a standard broiler chicken diet with 200 g/kg peach pomace meal.

^b^ PPM = peach pomace meal.

^c^ SCT = Soluble condensed tannins.

^d^ iCT = Insoluble condensed tannins.

^e^ TSP = Total soluble phenolics.

^f^ TiPh = Total insoluble phenolics.

### 2.4 Animal Husbandry and Research Layout

A total of 600 1‐day‐old, unsexed Ross 308 broiler chickens were supplied by Chicken Ranch Animal Feeds & Agricultural Supplies (PTY) LTD (Onderstepoort, Gauteng, South Africa). The birds were given Phenix Stresspac (vitamins and electrolytes) for 3 days through drinking water and a standard commercial starter diet until 4 days old. At 5 days old, the chicks were vent sexed, and the males (*n* = 280) were equally placed into 28 pens (determined using G*Power 3.1.9.7 for Windows), to which the four experimental diets were randomly distributed, producing seven replicates per treatment in a completely randomized design. The experimental units were pens (3.7 m length × 1.5 m width × 2.0 m height) with sunflower husk‐covered floors, and each pen accommodated 10 chicks. Each pen contained feeding and drinking troughs cleaned daily and replenished with fresh feed and water. The feeding trial was conducted under natural lighting. In the first 2 weeks, infrared electric lights maintained a temperature of 34°C, later decreased by 2°C biweekly until reaching 28°C. The building was ventilated by opening the curtains at 06:00 and closing them at 18:00.

### 2.5 Data Collection and Laboratory Investigations

Data collection, including mortality, lasted from 7 to 35 days of age. The birds' initial body weight (IBW; 140 ± 0.07 g) was determined on day 7. Daily measurements of average feed intake (**FI**) per pen involved calculating the variance between the provided feed and refusals. Subsequently, body weight per pen was determined using a digital scale (ADAM scale, readability of 0.5–2 g, Adam Equipment S.A. PTY, Johannesburg, South Africa) until day 35 to ascertain the average body weight gain (**BWG**) per week. The feed conversion ratio (**FCR**) was calculated as the ratio of the FI to the BWG data. The incidence of mortality was documented as a percentage and utilized to correct the growth performance data.

On the 33rd day, blood samples were collected from the brachial vein of two representatives of the average weight per pen and never returned to their respective pens to avoid cannibalism. The blood collection was conducted using 23‐gauge needles and 5 mL syringes, and the obtained samples were promptly transferred into whole blood and serum tubes. Hematological parameters (hematocrit, white cell count, platelet, lymphocyte, heterophil, and monocyte) were determined using an automated IDEXX LaserCyte Hematology Analyzer (IDEXX Laboratories Pty. S.A., Midrand, South Africa). Additionally, serum biochemical parameters (glucose, symmetric dimethylarginine, calcium, inorganic phosphate, total protein, albumin, globulin, albumin: globulin ratio, alanine transaminase, alkaline phosphatase, gamma‐glutamyl transferase, lipase, amylase, cholesterol, bilirubin, and creatinine) were analyzed using an automated IDEXX Vet Test Chemistry Analyzer (IDEXX Laboratories Pty. S.A., Midrand, South Africa).

At 35 days of age, all birds were weighed to establish their final body weight (**FBW**), and four representatives of the average weight per pen were electrically stunned and killed at a certified abattoir by severing the jugular vein with a sharp knife. The carcasses were labeled according to the experimental group with distinct colored wool fiber wrapped around the foot. The carcasses were weighed soon after dressing and evisceration to ascertain hot carcass weight (**HCW**), then refrigerated for 24 h at 4°C to measure cold carcass weight (**CCW**) without the viscera (gizzard, liver, and intestines). The carcass yield was determined by dividing the CCW by the FBW. The carcasses were then chopped to determine the weights of the wings, drumsticks, thighs, breasts, and viscera (gizzard, liver, and intestines) and expressed as a percentage of the CCW.

Meat pH was assessed at 24 h postmortem for all the carcasses utilizing a Corning Model 4 pH‐temperature meter equipped with an Ingold spear‐type electrode (Ingold Messtechnik AG, Udorf, Switzerland). The pH meter underwent calibration after every treatment group measurement using standard solutions (pH 4, 7, and 10). Subsequently, the color coordinates (lightness, L*; redness, a*; and yellowness, b*) were promptly determined using a Minolta color guide (BYK‐Gardener GmbH, Geretsried, Germany), which was calibrated by the manufacturer's guidelines. The color outcome represents the average of three measurements taken from different sections of the breast muscle.

Breast meat samples (*n* = 5 per replicate) were measured for water holding capacity, cooking loss, and drip loss according to methods previously described by Whiting and Jenkins (1981), Honikel (1998), and Zhang et al. (2017), respectively. The shear force (**SF**), an indicator of meat tenderness, was assessed by shearing freshly cut breast meat samples (*n* = 5 per replicate) using a Meullenet‐Owens Razor Shear Blade attached to a Texture Analyzer (TA‐XT plus, Stable Micro Systems, Surrey, UK).

### 2.6 Statistical Analysis

All evaluated data underwent normality assessments utilizing the normal option in the Proc Univariate statement in SAS before being analyzed using one‐way ANOVA (SAS 2013). The pen was considered the experimental unit for growth performance, while individual birds were the experimental units for blood parameters, carcass and visceral traits, and meat quality. Weekly recorded measurements (FI, BWG, and FCR) were subjected to repeated measures analysis in the general linear model (SAS 2013). This analysis examined the interactive effects of diet and week (bird age). Significance was determined at *p <* 0.05, and the probability of difference option within the LSMEANS statement in SAS was employed to distinguish among treatments.

## 3 Results

### 3.1 Growth Performance

Throughout the research period, five birds died, resulting in a mortality rate of 1.79%. Five birds died between days 28 and 35: one in the CON and PPM150, and three in the PPM200 group.

The repeated measures analysis indicated a significant diet × week (bird age) on average weekly feed intake (FI; *p* = 0.020) and feed conversion ratio (FCR; *p* = 0.033), but not on body weight gain (BWG; *p* = 0.284). Table 3 indicates significant dietary effects (*p <* 0.05) only on FI during days 7–14 and 29–35. On days 7–14 and 29–35, birds reared on PPM200 had higher (*p <* 0.05) FI than CON but did not vary with birds fed PPM100 and PPM150 (*p >* 0.05).

**TABLE 3:** Growth performance (g/bird, unless otherwise specified) of Ross 308 chickens fed dietary peach pomace meal.

Parameters^b^	Treatments^a^					Significance
	CON	PPM100	PPM150	PPM200	SEM^c^	*P*‐_GLM_
IBW (day 7)	141	138	138	142	3.46	0.780
FBW (day 35)	2069	2201	2108	2098	52.0	0.341
Overall BWG	1928	2063	1969	1956	49.8	0.284
Average weekly feed intake						
Days 7–14	418^e^	515^de^	517^de^	529^d^	25.5	0.025
Day 15–21	580	629	605	643	23.0	0.266
Days 22–28	801	771	750	810	28.7	0.457
Day 29–35	1138^e^	1233^de^	1228^de^	1243^d^	25.5	0.036
Average weekly FCR (g:g)						
Days 7–14	1.70	1.97	1.97	2.05	0.09	0.108
Days 15–21	1.38	1.54	1.52	1.51	0.07	0.368
Days 22–28	1.34	1.16	1.22	1.30	0.05	0.095
Days 29–35	1.72	1.64	1.78	1.94	0.08	0.085
Mortality (%; 7–35 days)	0.20	0.00	0.20	0.60	0.25	0.395

^a^ Treatments: CON = a standard broiler chicken diet without peach pomace meal; PPM100 = a standard broiler chicken diet with 100 g/kg peach pomace meal; PPM150 = a standard broiler chicken diet with 150 g/kg peach pomace meal; PPM200 = a standard broiler chicken diet with 200 g/kg peach pomace meal.

^b^ Parameters: IBW = initial body weight; FBW = final body weight; BWG = body weight gain; FCR = feed conversion ratio.

^c^ SEM: standard error of the mean.

^d,e^ Means in a row having distinct superscripts vary significantly (*p* < 0.05).

### 3.2 Blood Parameters

Table 4 shows the blood parameters of birds reared on diets containing incremental PPM levels. The dietary inclusion of PPM did not induce significant effects (*p >* 0.05) on all blood parameters measured except lymphocyte count and bilirubin concentration. Birds reared on PPM100, PPM150, and PPM200 exhibited comparable lower (*p <* 0.05) lymphocyte counts and bilirubin concentrations than CON.

**TABLE 4:** Blood parameters of Ross 308 chickens fed dietary peach pomace meal.

Parameters	Treatments^a^					Significance
	CON	PPM100	PPM150	PPM200	SEM^b^	*P*‐_GLM_
Hematocrits (%)	27.4	26.6	28.0	30.4	1.74	0.469
White cell counts (× 10^9^/L)	16.9	12.7	14.1	12.9	1.73	0.333
Platelets (× 10^9^/L)	21.2	18.7	27.9	22.1	5.01	0.619
Heterophils (× 10^9^/L)	10.0	8.72	8.76	8.65	1.25	0.837
Lymphocytes (× 10^9^/L)	6.51^c^	3.14^d^	3.75^d^	3.79^d^	0.68	0.014
Monocytes (× 10^9^/L)	0.57	0.69	0.86	0.44	0.26	0.694
Glucose (mm/L)	11.3	11.0	10.6	11.3	0.76	0.912
Symmetric dimethylarginine (μg/dL)	4.80	2.40	6.00	6.20	1.48	0.280
Calcium (mm/L)	2.35	2.33	2.59	2.37	0.10	0.295
Inorganic phosphate (mm/L)	1.73	2.08	2.11	1.74	0.13	0.079
Total protein (g/L)	26.4	28.6	25.8	26.6	1.82	0.723
Albumin (g/L)	11.2	11.6	11.2	11.4	0.71	0.973
Globulin (g/L)	15.2	17.0	14.6	15.2	1.19	0.537
Albumin: globulin ratio	0.74	0.69	0.78	0.75	0.03	0.287
Alanine transferase (U/L)	3.00	3.60	3.40	3.80	0.37	0.495
Gamma‐glutamyl transferase (U/L)	21.0	17.0	21.6	18.6	2.17	0.431
Amylase (U/L)	401	449	349	534	102	0.630
Lipase (U/L)	5.80	7.20	9.00	7.60	1.34	0.431
Cholesterol (mm/L)	3.22	3.06	2.68	2.88	0.21	0.340
Bilirubin (μmol/L)	1.60^c^	1.00^d^	1.00^d^	1.00^d^	0.12	0.006
Creatinine (μmol/L)	2.60	2.00	2.40	2.80	0.58	0.795

^a^ Treatments: CON = a standard broiler chicken diet without peach pomace meal; PPM100 = a standard broiler chicken diet with 100 g/kg peach pomace meal; PPM150 = a standard broiler chicken diet with 150 g/kg peach pomace meal; PPM200 = a standard broiler chicken diet with 200 g/kg peach pomace meal.

^b^ SEM: standard error of the mean.

^c,d^ Means in a row having distinct superscripts vary significantly (*p* < 0.05).

### 3.3 Carcass Traits and Organ Weights

Table 5 indicates no significant (*p >* 0.05) dietary effects on all the carcass traits and organ weights.

**TABLE 5:** Carcass traits and organ weight (g/100 g CCW, unless specified otherwise) of Ross 308 chickens fed dietary peach pomace meal.

Parameters^b^	Treatments^a^					Significance
	CON	PPM100	PPM150	PPM200	SEM^c^	*P*‐_GLM_
Carcass yield (%)	76.4	76.9	76.9	75.9	1.68	0.435
HCW (g)	1580	1715	1631	1646	69.9	0.594
CCW (g)	1562	1692	1622	1593	74.0	0.629
Breast	26.6	25.5	24.5	24.7	0.22	0.242
Drumstick	6.72	7.09	6.91	7.16	0.44	0.154
Thigh	9.92	9.34	9.37	8.22	0.73	0.050
Wing	5.76	5.91	5.79	6.09	0.34	0.229
Gizzards	2.59	2.39	2.47	2.32	0.20	0.136
Intestines	7.94	7.33	7.65	7.66	0.56	0.505
Liver	3.63	3.25	3.02	3.27	0.35	0.203

^a^ Treatments: CON = a standard broiler chicken diet without peach pomace meal; PPM100 = a standard broiler chicken diet with 100 g/kg peach pomace meal; PPM150 = a standard broiler chicken diet with 150 g/kg peach pomace meal; PPM200 = a standard broiler chicken diet with 200 g/kg peach pomace meal.

^b^ Parameters: HCW = hot carcass weight; CCW = cold carcass weight.

^c^ SEM: standard error of the mean.

### 3.4 Meat Quality

The inclusion of dietary PPM had an influence (*p <* 0.05) on the meat L* and a* (Table 6). Birds on PPM150 and PPM200 had comparably higher (*p <* 0.05) L* than the PPM100; however, PPM100 did not vary from CON (*p >* 0.05). Furthermore, PPM100 had a higher (*p <* 0.05) a* than the CON and PPM150; however, CON did not differ (*p >* 0.05) from PPM200.

**TABLE 6:** Meat quality parameters of Ross 308 chickens fed dietary peach pomace meal.

Parameters^b^	Treatments^a^					Significance
	CON	PPM100	PPM150	PPM200	SEM^c^	*P*‐_GLM_
pH	5.87	5.87	5.84	5.82	0.09	0.531
L*(lightness)	45.8^de^	44.2^e^	47.3^d^	47.3^d^	0.77	0.019
a* (redness)	3.55^e^	5.49^d^	3.30^e^	3.83^de^	0.42	0.003
b* (yellowness)	2.88	2.35	2.00	2.72	0.33	0.242
Cooking loss (%)	26.0	28.4	25.6	26.7	2.07	0.252
Drip loss (%)	7.82	10.0	9.54	9.37	0.67	0.126
Water holding capacity (%)	81.2	79.7	82.9	77.9	2.53	0.545
Shear force (N)	4.40	4.98	4.98	4.68	0.63	0.899

^a^ Treatments: CON = a standard broiler chicken diet without peach pomace meal; PPM100 = a standard broiler chicken diet with 100 g/kg peach pomace meal; PPM150 = a standard broiler chicken diet with 150 g/kg peach pomace meal; PPM200 = a standard broiler chicken diet with 200 g/kg peach pomace meal.

^c^ SEM: standard error of the mean.

^d,e^ Means in a row having distinct superscripts vary significantly (*p* < 0.05).

## 4 Discussion

The incremental dietary PPM did not have a notable impact on the mortality of the birds, with less than 2% mortality observed throughout the study, which was anticipated. This finding is consistent with the observation of Sosnówka‐Czajka et al. (2023), who spotted no significant variations in Ross 308 chickens' mortality when 3% apple, cherries, and strawberry pomace were included in their diet. Earlier studies have shown that the effects of fruit pomace on bird growth performance can vary from enhancement to poor growth performance. Surprisingly, FI in birds fed PPM diets was not suppressed, while the FCR did not vary despite their higher crude fiber contents. This result contrasts with the reports by Kumanda et al. (2019); the authors observed that incorporating 7.5% dietary red grape pomace decreased the FI in Cobb 500 chickens. Consistent with our findings is the report by Erinle et al. (2022), who noted that including 2.5% dietary grape pomace did not reduce overall FI in Cobb 500 chickens. Our study indicated that PPM200 enhanced FI (days 7–4 and 29–35) without decreasing overall BWG compared to CON, implying that the PPM fiber did not impair nutrient digestibility or availability. Similarly, Colombino et al. (2020) reported no difference in BWG of Ross 308 chickens reared on 6% dietary apple, blackcurrant, or strawberry pomace. The comparable FCR among the treatments implies that the higher crude fiber content of PPM100, PPM150, and PPM200 may not have compromised the bioavailability of nutrients, which was not anticipated. Furthermore, the observed comparable FCR among dietary treatments could be attributed to the multifaceted effects of dietary fiber from PPM (Table 2) on FI, nutrient utilization, and gut health regulation (Nassar et al. 2019). Further analyses exploring the specific fiber fractions, their fermentation kinetics, and their interactions with other diet components would provide valuable insights into the mechanisms underlying the observed results.

Blood parameters can accurately evaluate poultry biological function and clinical condition (Egbu et al. 2022; Erinle et al. 2022). In the present study, dietary PPM reduced the lymphocyte count and bilirubin concentration. In contrast to our findings, Kumanda et al. (2019) showed that dietary grape pomace did not yield any notable impact on lymphocyte count or bilirubin concentration. The lack of significant impacts by Kumanda et al. (2019) might stem from the documented lower levels (75 g/kg) of grape pomace incorporated into the diet. Lower bilirubin concentrations among the PPM groups may indicate improved liver function or reduced red blood cell breakdown (Harr 2002), potentially influencing the immune response. Alanine transferase and gamma‐glutamyl transferase are enzymes primarily associated with liver function. Their comparable levels among treatments further suggest that the dietary PPM did not compromise liver health. In contrast, Das et al. (2020) reported reduced alanine transferase levels in 21‐day‐old Cobb 500 broiler chickens reared on dietary 2% blueberry pomace. The authors implied that the reduced alanine transferase levels were due to the anthocyanins present in blueberry pomace. However, the precise biological mechanism by which phenols influence blood parameters remains unclear. Although gut microbial populations were not assessed in this study, the combination of dietary fiber and phenolic compounds in PPM may modulate microbiota composition, influencing nutrient utilization and immune responses.

Among the indicators of the economic sustainability of a poultry business is the quantity of high‐quality meat produced. The dietary PPM did not enhance the carcass traits or the visceral organs, with comparable results observed in prior investigations (Aditya et al. 2018; Kumanda et al. 2019). The observed comparable visceral organ values suggest that dietary PPM failed to trigger physiological adaptability measures in the birds. In theory, it was anticipated that the chickens reared on dietary PPM would have elongated intestines as an adaptive response to utilize fiber and improve the digestive process efficiently. We attribute this to the levels of PPM inclusion that might not have provided a significant increase in dietary fiber content to elicit a noticeable adaptive response in the chickens' digestive physiology, differently than anticipated.

We anticipated alterations in the breast meat color because dietary PPM is a natural source of pigments like carotenoids and anthocyanins (Liu et al. 2018; Vulić et al. 2022). According to Dlamini et al. (2023), the meat color holds significance for consumers as a key factor influencing perceptions of quality and shelf life. Kirkpinar et al. (2001) posited that carcasses with reduced L* and increased a* and b* scores suggest a superior and more desirable color. In this study, breast meat a* increased, which corroborates the findings of Kumanda et al. (2019). Dietary PP contains phenolics (Table 2), which can impact meat color by influencing myoglobin and lipid oxidation rates. In contrast with our study, Colombino et al. (2020) demonstrated no changes in the breast meat color of chickens fed 6% dietary apple, blackcurrant, or strawberry pomace. Similarly, in studies by Jonathan et al. (2021), dietary red grape pomace at 60 g/kg did not influence the meat color. These contrasting color responses likely reflect the concentration‐dependent redox behavior of phenolic compounds. At PPM100, phenolics such as chlorogenic acid and catechin can act as chain‐breaking antioxidants, scavenging lipid radicals and protecting myoglobin from oxidative discoloration, enhancing a* in breast muscle (Amaral et al. 2018). However, at PPM150 and PPM200, excess phenolics may undergo auto‐oxidation or redox cycling in the presence of transition metals, generating reactive quinone intermediates and hydrogen peroxide that promote lipid and pigment oxidation (pro‐oxidant action) (Sierżant et al. 2023). Such pro‐oxidant activity can accelerate metmyoglobin formation and lipid peroxidation, offsetting initial color benefits. To ascertain this mechanism, future work should quantify oxidative‐stress markers such as TBARS and protein carbonyls alongside breast meat color metrics. Investigations on the utilization of fruit pomace have yielded varying outcomes, particularly regarding physiological and meat quality measures. This may be owing to the fluctuating amounts of nutraceuticals found in different fruit pomaces, which are influenced by nutrient composition, processing, cultivar, soil conditions, and climate (Van Niekerk et al. 2020; Vulić et al. 2022; Yao et al. 2021). The total soluble phenol concentration in the PPM used in this trial is 15.8 g TAE/g, which differs from the 16.43 g TAE/g documented in grape pomace (Van Niekerk et al. 2020), 6.31 mg GAE/g found in raspberry pomace (Yao et al. 2021), and 8.43 in apple, 26.7 in blackcurrant, and 28.9 mg/g GAE/g in strawberry pomace (Das et al. 2020).

## 5 Conclusion

The dietary inclusion of 100 g/kg of peach pomace meal did not compromise broiler chicken production outcomes or meat quality. These results offer practical implications for poultry producers, feed manufacturers, and policymakers by highlighting the feasibility of using peach pomace meal as a feed ingredient in broiler diets. This has the potential to promote sustainability, reduce production costs, ensure meat quality, and expand the utilization of alternative feed resources within the poultry industry.

## Author Contributions

**Tshegofatso Moroka:** data curation (equal), methodology (equal), validation (equal). **Kgaogelo Phineas Meso:** investigation (equal), methodology (equal), resources (equal), validation (equal). **Lebogang Ezra Motsei:** conceptualization (equal), project administration (equal), resources (equal), supervision (equal), validation (equal), writing – review and editing (equal). **Chidozie Freedom Egbu:** conceptualization (equal), data curation (equal), formal analysis (equal), methodology (equal), software (equal), supervision (equal), validation (equal), visualization (equal), writing – original draft (equal), writing – review and editing (equal).

## Disclosure

Declaration of generative AI and AI‐assisted technologies in the writing process: The authors did not use any artificial intelligence‐assisted technologies in the writing process.

## Conflicts of Interest

The authors declare no conflicts of interest.

## Data Availability

The data that support the findings of this study are available from the corresponding author upon reasonable request.
